# Extrinsic Factors Contributing to Achilles Tendon Ruptures During NFL Games From 2010-2023: A Retrospective Analysis

**DOI:** 10.1177/23259671261458572

**Published:** 2026-07-16

**Authors:** Kelan Queenan, Spencer DeMedal, Hasala Rannulu, Preston Rogers, Luke Jordan, Sreeram Ravi, Scott Lynch, Robert A. Gallo

**Affiliations:** † Penn State College of Medicine, Hershey, Pennsylvania, USA; ‡ Department of Orthopaedic Surgery and Rehabilitation, Penn State Milton S. Hershey Medical Center, Hershey, Pennsylvania, USA; Investigation performed at Penn State College of Medicine, Milton S. Hershey Medical Center, Hershey, Pennsylvania, USA

**Keywords:** Achilles rupture, Achilles tendon tear, football, National Football League

## Abstract

**Background:**

Achilles tendon ruptures are serious injuries that significantly impair performance and career longevity among professional football players. The incidence of these injuries in National Football League (NFL) athletes has increased over the past decade. Given the sport's high physical demands and the potential for long-term functional limitations, understanding the extrinsic factors contributing to Achilles tendon tears is crucial.

**Hypothesis/Purpose:**

The purpose of this study was to evaluate the role of extrinsic factors in the incidence of Achilles tendon ruptures among NFL players. Playing surface, timing within the season, quarter of play, and days of rest prior to the tear were examined. It was hypothesized that Achilles tendon tears would occur more frequently on artificial turf, during the fourth quarter of games, and in the latter half of the regular season.

**Study Design:**

Descriptive epidemiology study.

**Methods:**

Publicly available NFL injury data from 2010 to 2023 were retrospectively reviewed. Injury reports and related articles from major sports media sources were used to identify players placed on the injured reserve due to Achilles tendon ruptures sustained during official games. Descriptive statistics summarized player characteristics. Game-level analyses were conducted using analysis of variance and Pearson chi-square testing, while player-level comparisons utilized 1-sample *t* tests and chi-square tests of independence. *P* < .05 was considered statistically significant.

**Results:**

Of 1,477 players initially identified with ankle injuries, 103 players (103 ankles) sustained complete Achilles tendon ruptures across 101 games. The incidence of Achilles tears was highest in 2023 (n = 14 [13.59%]) and 2020 (n = 13 [12.62%]). The highest weekly incidence of tendon tear occurred in week 4 of the regular season (n = 13 [12.62%]). No significant differences in injury patterns were observed based on playing surface (2.68% grass vs 1.88% turf; χ^2^ = 0.18; *P* = .67), game quarter (*P* = .18), or season timing (first half 57.42% vs. second half 27.18% of the season, *P* = .07).

**Conclusion:**

Our study demonstrated that Achilles tendon ruptures in NFL players have increased in frequency since 2020, without significant associations between playing surface, game timing, or season progression. Identifying modifiable external risk factors may help guide injury prevention strategies and optimize player training and conditioning.

Achilles tendon tears account for a substantial proportion of lower extremity tendinous injuries.^2^ Although the Achilles tendon is the strongest tendon in the body, repetitive overuse can result in a spectrum of pathology ranging from tendinitis to acute rupture.^12^ Spontaneous rupture is a serious injury that typically occurs due to sudden, excessive loading of the tendon or an underlying degenerative process.^12^ These injuries most often result from forced plantarflexion or rapid dorsiflexion of a plantar-flexed foot.^3,12^

Achilles tendon ruptures are frequently season-ending and result in significant time lost from competition among collegiate and professional athletes.^4^ Approximately 30% to 40% of professional athletes do not return to play after these injuries.^10,20^ In addition to physical limitations, athletes may experience psychological effects, including fear of reinjury, which can further impact performance.^11^ In the National Football League (NFL), the mean time to return to play after an Achilles tendon rupture is approximately 12 months, with only about 60% of players successfully returning to competition.^20^ Notably, the incidence of Achilles tendon ruptures in NFL players has increased over the past decade, particularly within the last 5 years.^1^

Despite growing concern, data remains limited on the external factors contributing to lower extremity injuries in NFL players, and even fewer studies have specifically examined Achilles tendon ruptures.^13^ One study reported a 16% higher incidence of lower extremity injuries on artificial turf compared with natural grass, although Achilles-specific outcomes were not evaluated.^9^ Furthermore, there is a paucity of evidence examining factors such as playing surface, game timing, season timing, and player position in relation to Achilles tendon ruptures.

Understanding these factors is critical, as Achilles tendon ruptures are severe injuries that often require surgical repair, prolonged rehabilitation, and substantial time away from competition, with many athletes unable to return to their previous level of performance. Identifying modifiable risk factors may help inform injury-prevention strategies, including optimizing playing surfaces, adjusting training and workload throughout the season, and targeting conditioning for high-risk player groups. These findings may also guide decision making among team physicians, athletic trainers, league officials, and stadium management regarding athlete safety and playing conditions.

The purpose of this study was to evaluate extrinsic risk factors associated with Achilles tendon ruptures in NFL players. We hypothesized that ruptures would occur more frequently on artificial turf, during the fourth quarter of games, and in the latter half of the regular season.

## Methods

### Search Strategy

A retrospective observational study was conducted using publicly available NFL injury reports. Injury data were obtained from Columbia Broadcasting System Sports (CBS Sports), Entertainment and Sports Programming Network (ESPN), and NFL.com to identify players placed on injured reserve, a designation for athletes unable to participate for an extended period due to injury. NFL players with reported Achilles tendon, ankle, or lower extremity injuries between the 2010 and 2023 seasons were included.

### Search Criteria

The initial player list was refined to include only confirmed complete Achilles tendon tears. Six medical students independently performed data extraction, with diagnoses verified across multiple sources. To standardize game conditions, only complete tears occurring during preseason, regular season, and postseason games were included; international regular-season games were also included, with the playing surface recorded for each. Tears sustained during organized team activities, practices, scrimmages, summer training, or the offseason were excluded, as were partial tears, strains, and tendinopathies.

To compare the incidence of complete Achilles tendon tears with the total number of NFL games, games with at least 1 tear were counted rather than the total number of affected players. Two games included multiple injuries (week 1 of the 2014 season and week 3 of the 2023 season). No bilateral Achilles tendon tears were identified.

### Data Extraction

#### Player-Level Data

Player characteristics, including age and position at the time of injury, were collected. For each position, the mean age of all rostered players (injured and uninjured) was calculated for each season from 2010 to 2023 using publicly available data; these values were then averaged to generate an overall mean age per position.

Offensive positions included quarterback, running back, wide receiver, tight end, center, offensive guard, and offensive tackle. Defensive positions included linebacker (inside and outside), safety (strong and free), cornerback, defensive end, and defensive tackle.

Player rest time was defined as the number of days between the last game played and the Achilles tendon tear. Given the potential career-ending nature of these injuries, the time from injury to retirement was also recorded, when applicable. Tear laterality and whether the injury represented a primary rupture or retear were documented. Complete data were available for all variables of interest.

#### Game-Level Data

Game information included week of the season, game quarter, stadium, location, and playing surface at the time of injury. Stadium playing surface (turf or grass), specific manufacturer, and presence or absence of a dome were tracked over the 14-year study period (Appendix 1).

Lambeau Field, home of the Green Bay Packers, utilized GrassMaster hybrid turf from 1957 to 2017 and transitioned to natural Kentucky bluegrass reinforced with SIS Grass polyethylene fibers from 2018 onward. Given evidence that this hybrid system is predominantly composed of natural grass, Lambeau Field was classified as a grass surface for the purposes of this study.

Nissan Stadium, home of the Tennessee Titans, transitioned from natural grass to artificial turf in the fall of 2023; therefore, games played at Nissan Stadium during the 2023 season were classified as turf.

The total number of games played on turf and grass each week of the season was also recorded.

### Data Analysis

Descriptive statistics were used to summarize player characteristics. A 1-sample *t* test was performed to compare the mean age at the time of injury by position with the corresponding mean NFL position age over the 14-year study period. Confidence intervals were calculated at the 95% confidence level (CL) to estimate parameter ranges.

A Pearson chi-square test was used to compare the number of games with Achilles tendon tears between playing surfaces (turf vs. grass). One-way analysis of variance was used to assess differences in Achilles tendon tear occurrence across weeks of the season. A chi-square test of independence was performed to evaluate the association between reinjury and playing surface.

Reported findings represent observed frequencies rather than true incidence rates. Statistical significance was defined as *P* < .05.

## Results

A total of 1,477 players were identified in the initial search of NFL players with any reported ankle injury between the 2010 and 2023 seasons. After full review, 103 players with 103 confirmed Achilles tendon tears were included. Overall, 101 of 4,600 NFL games analyzed (2.26%) involved at least 1 Achilles tendon tear.

### Player Characteristics

Players with Achilles tendon tears from the 2010-2023 seasons had a mean age of 27.26 ± 3.43 years. No NFL position demonstrated a statistically significant difference between age at the time of injury and the mean position age in the NFL over the study period (all *P* > .05) (Table 1).

Among retired players, the mean time from Achilles tendon tear to retirement was 2.40 ± 1.93 years (n = 58 [56.31%]). Overall, 100 injuries (97.09%) were primary Achilles tendon tears, while 3 (2.91%) were retears of the ipsilateral Achilles tendon.

### Achilles Tendon Tears by NFL Season

From 2010 to 2023, the highest number of Achilles tendon ruptures occurred during the 2023 season (n = 14 [13.59%]). The 2017, 2020, and 2021 seasons also had high injury counts, with 10, 13, and 11 ruptures, respectively. Collectively, these 4 seasons accounted for 46.60% (48/103) of all Achilles tendon tears over the study period.

### Achilles Tendon Tears by Season Phase

Over the study period, 13 (12.62%) Achilles tendon tears occurred during preseason games. During the regular season, week 4 had the highest number of ruptures (n = 13 [12.62%]).

In total, 58 games (57.42%) in which an Achilles tear occurred took place during the first half of the regular season (weeks 1-8), compared with 28 games (27.18%) in the second half (weeks 9-18). Two tears (1.94%) occurred during the postseason (wild card and conference championship games). There was no significant difference in the proportion of games with Achilles tears between the first half (7.88 ± 6.76) and the second half of the regular season (6.68 ± 4.31; *P* = .07) (Table 2).

**Table 2:** Analysis of Variance for Frequency of Games with Achilles Tendon Tears by Season Phase*^*a*^*

Game Week	N	Mean	Standard Deviation	Lower 95% CL for Mean	Upper 95% CL for Mean
Hall of fame/preseason	13	6.90	1.37	5.92	7.88
Regular season, weeks 1-8	58	7.88	6.76	2.38	4.16
Regular season, weeks 9-18	28	6.68	4.31	0.80	1.87
Postseason	2	8.50	2.12	–10.56	27.56

a CL, confidence level.

**Table 1:** One-Sample *t*-Test for Age of Achilles Tendon Tears Compared With the Mean Age of NFL Positions*^*a*^*

	NFL Position	No. of Players	Age at Achilles Tear	Age of Average NFL Position	*P*	95% CI
Offense	Quarterback	2	37 ± 2.82	28.99 ± 0.82	.156	11.59-62.41
	Running back	11	25.55 ± 3.05	25.73 ± 0.91	.845	23.50-27.59
	Wide receiver	6	26.33 ± 3.01	26.69 ± 0.36	.783	23.17-29.49
	Tight end	10	26.40 ± 3.63	26.50 ± 0.43	.932	23.81-28.99
	Center	2	32.50 ± 6.36	27.46 ± 1.97	.464	24.68-89.68
	Offensive guard	2	28 ± 0.71	26.50 ± 1.23	.375	15.29-40.71
	Offensive tackle	5	27.20 ± 3.56	26.50 ± 0.71	.683	22.78-31.62
Defense	Linebacker, including inside, outside	23	27.22 ± 2.90	26.89 ± 0.59	.589	25.98-28.45
	Safety, including strong, free	14	28.08 ± 2.10	26.66 ± 0.53	.337	24.81-27.35
	Cornerback	14	26.93 ± 2.84	26.40 ± 0.49	.499	25.29-28.57
	Defensive end	10	28.30 ± 3.02	26.99 ± 0.41	.203	26.14-30.46
	Defensive tackle	4	30.25 ± 4.57	26.47 ± 0.79	.197	22.97-37.53

a Data are presented as mean ± SD. NFL, National Football League.

Among injured players, the mean time between the previous game and injury was 7.38 ± 2.29 days (n = 97). Six players sustained an Achilles tendon tear in their first game of the season (5.82%).

**Figure 1.**
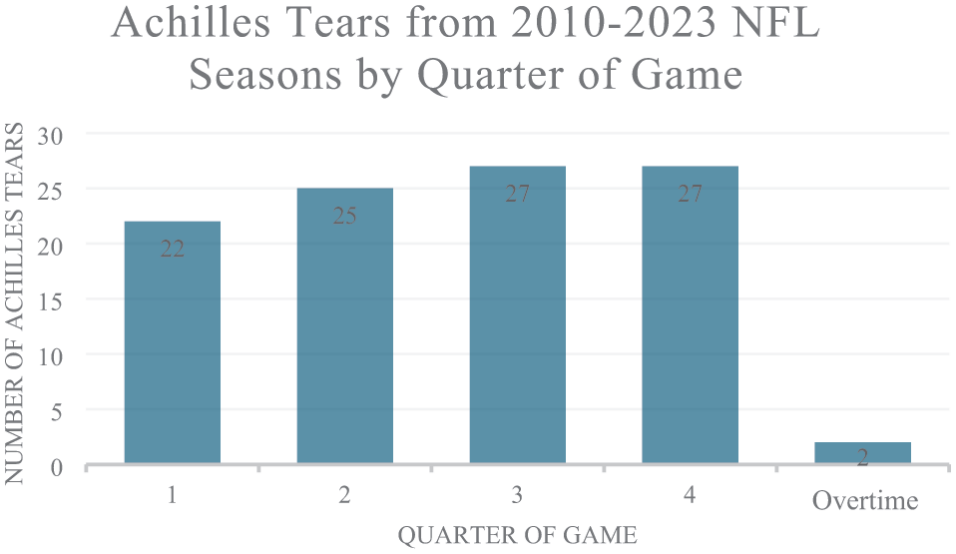
Achilles tendon tears by the quarter of the game.

Across games, 47 (45.63%) tears occurred in the first half (first or second quarter), while 54 (52.42%) occurred in the second half (third or fourth quarter). This difference was not statistically significant (χ^2^ = 4.84; *df* = 3; *P* = .18). Two injuries (1.94%) occurred in overtime (Figure 1).

### Achilles Tendon Tears by Playing Surface

Of 2,050 games on grass, 55 (2.68%) had at least 1 Achilles tendon tear, compared with 48 of 2550 games on turf (1.88%). There was no statistically significant association between playing surface and the presence of an Achilles tendon tear (χ^2^ = 0.18; *P* = .67) (Table 3).

**Table 3:** Incidence of Achilles Tendon Tears by Playing Surface From 2010 to 2023

Playing Surface	Games With Achilles Tear, N (%)	Total Games, N	χ^2^ (*df* = 1)	*P*
Grass	55 (2.68)	2,050	0.18	.67
Turf	48 (1.88)	2,550		
Total	103 (2.24)	4,600		

## Discussion

Our study demonstrated that the incidence of Achilles tendon ruptures in the NFL has increased since 2020. Although not statistically significant, a greater proportion of Achilles tendon tears occurred in the first half of the season compared with the second half. No player position demonstrated a younger mean age at injury than the mean age of NFL players at that position. Additionally, the incidence of Achilles tendon tears did not differ significantly between natural grass and artificial turf.

Overall, our findings suggest an increasing incidence of Achilles tendon ruptures among NFL players, with a notable rise beginning in 2020. While the exact cause of this increase remains unclear, one proposed explanation is the impact of the COVID-19 2019 pandemic, during which reduced in-person training opportunities and altered preseason structures may have led to inadequate conditioning.^1,7^ Notably, this increased incidence persisted even after a return to more typical training and competition schedules.^15^

Deconditioning may increase tendon loading and susceptibility to injury upon return to high-intensity activity, thereby elevating the risk of rupture. The observed trend toward a higher proportion of injuries in the first half of the season may also be multifactorial, potentially reflecting changes in preseason preparation, roster turnover, and the reduction in preseason games from 4 to 3, which may have altered the early-season workload and increased the risk of overexertion. Further investigation is warranted to elucidate these associations better.

Tendon fatigue is a well-established risk factor for tendon injury. It is associated with microstructural damage, reduced tendon stiffness, and impaired capacity to withstand repetitive loading, all of which predispose the tendon to failure under stress.^6^ In cadaveric models, Achilles tendons exposed to cyclic fatigue loading have demonstrated increased ultrasound echogenicity changes and a higher likelihood of failure, suggesting that fatigue-related damage is a measurable precursor to rupture.^16^

This may help explain why a greater proportion of Achilles tendon ruptures occurred during the second half of games, potentially reflecting cumulative fatigue over the course of play that contributes to injury risk. However, our findings also demonstrated a higher proportion of ruptures in the first half of the NFL season, suggesting that acute fatigue (over hours of play), rather than chronic fatigue (over weeks to months), plays a more prominent role in rupture risk.

There has been ongoing debate regarding the role of the playing surface in Achilles tendon injuries, particularly in high-impact sports that require rapid acceleration and directional changes. Several studies suggest that synthetic turf may increase injury risk due to reduced cleat release, which can lead to higher peak torque and increased rotational stiffness at the shoe-surface interface.^8,9,13,17,18^ However, our results demonstrated no significant difference in Achilles tendon tear incidence between playing surfaces among NFL players.

It is likely that cleat design and the shoe-surface interface contribute to the risk of injury. Although the impact of cleat design on peak torque and rotational stiffness remains poorly characterized, certain footwear models have been shown to alter rotational traction significantly.^5,18^ Cleats with increased rotational stiffness may elevate Achilles tendon loading by increasing ankle joint torque and axial talar rotation during external rotation of the foot. This mechanism may place increased strain on the Achilles tendon, and repetitive high-torque movements, such as those encountered in football, may contribute to overuse, tendon degeneration, and eventual rupture.^3,19^ In addition, stiffer cleats and those with less compliant soles may further increase tendon strain and injury risk.^6,14^

Although Achilles tendon ruptures are likely multifactorial in etiology, further biomechanical research examining the interaction between cleat design and playing surface is needed to better elucidate their contribution to injury risk.

### Limitations

There are several limitations to this study. First, data were obtained from publicly available sources, which may be incomplete or subject to reporting inaccuracies, despite cross-verification across multiple platforms. Second, the analysis did not account for concomitant injuries or comorbid conditions at the time of Achilles tendon rupture, which may have influenced the risk of injury. Third, only tears that occurred during competitive games were included. Achilles tendon ruptures that occurred during practices or training activities were excluded, likely resulting in an underestimation of the total injury burden.

Fourth, when comparing age at injury by position to mean NFL position age, the mean position age was calculated by averaging publicly reported positional ages across seasons from 2010 to 2023, which does not account for year-to-year variation or allow for individual-level matching. Fifth, all synthetic and natural surfaces were grouped broadly, which may mask differences between specific turf systems or grass types.

Finally, games were analyzed as the unit of comparison (games with at least 1 Achilles tendon tear versus total games played). In 2 instances, multiple Achilles tendon tears occurred within a single game (week 1 of the 2014 season and week 3 of the 2023 season), which may have resulted in slight underrepresentation of total injury events. However, this is unlikely to impact the overall findings meaningfully.

## Conclusion

Our study demonstrated that Achilles tendon ruptures in NFL players have increased in frequency since 2020, without significant associations between playing surface, game timing, or season progression. Identifying modifiable external risk factors may help guide injury prevention strategies and optimize player training and conditioning.

## Appendix

**Appendix 1:** NFL Stadiums and Field Playing Surfaces From 2010 to 2023*^*a*^*

NFL Playing Surfaces by Stadium, 2010-2023					
Team	Location	Stadium	Playing Surface Manufacturer	Surface	Dome
Arizona Cardinals	Glendale, AZ	State Farm Stadium (2006-present) *^*b*^* Formerly University of Phoenix Stadium until 2018	Bermuda Grass	Grass	Yes, retractable
Atlanta Falcons	Atlanta, GA	Mercedes-Benz Stadium (2017-present) Georgia Dome (1992-2016)	Field Turf	Turf	Yes, retractable
Baltimore Ravens	Baltimore, MD	M&T Bank Stadium	Bermuda Grass (2016-present) Sportexe Turf (2010-2016)	*^*b*^*	No
Buffalo Bills	Orchard Park, NY	Highmark Stadium *^*b*^* Formerly Ralph Wilson Stadium and New Era Field	A-Turf Titan 50	Turf	No
Carolina Panthers	Charlotte, NC	Bank of America Stadium *^*b*^* Formerly Ericsson Stadium	FieldTurf (2021-present) Bermuda Grass (1996-2021)	*^*b*^*	No
Chicago Bears	Chicago, IL	Soldier Field	Bermuda Grass (2022-present) Kentucky Bluegrass (until 2021)	Grass	No
Cincinnati Bengals	Cincinnati, OH	Paycor Stadium *^*b*^* Formerly Paul Brown Stadium	Slit-Film Turf (2018-present) ACT Global Turf (2012-2017) FieldTurf (2004-2011) Grass (2000-2004)	Turf	No
Cleveland Browns	Cleveland, OH	FirstEnergy Stadium *^*b*^* Formerly Cleveland Browns Stadium	Kentucky Bluegrass (1999-present)	Grass	No
Dallas Cowboys	Arlington, TX	AT&T Stadium	Hellas Matrix Turf (2009-present)	Turf	Yes, retractable
Denver Broncos	Denver, CO	Empower Field at Mile High *^*b*^* Formerly Invesco Field, Sports Authority	Kentucky Bluegrass	Grass	No
Detroit Lions	Detroit, MI	Ford Field	Monofilament Field Turf (2023-present) Slit-Film Turf (2002-2022)	Turf	Yes
Green Bay Packers	Green Bay, WI	Lambeau Field	Kentucky Bluegrass with SIS fibers (2018-present) GrassMaster Hybrid (1957-2017)	Grass	No
Houston Texans	Houston, TX	NRG Stadium *^*b*^* Formerly Reliant Stadium	Hellas Matrix Turf (2015-present) Grass (2002-2015)	*^*b*^*	Yes, retractable
Indianapolis Colts	Indianapolis, IN	Lucas Oil Stadium	Slit-Film Turf	Turf	Yes, retractable
Jacksonville Jaguars	Jacksonville, FL	TIAA Bank Field *^*b*^* Formerly Jacksonville Municipal Stadium, Alltel Stadium, Everbank Field	Bermuda Grass	Grass	No
Kansas City Chiefs	Kansas City, MO	Arrowhead Stadium	Bermuda Grass	Grass	No
Las Vegas Raiders	Paradise, NV	Allegiant Stadium (2020-present) Oakland Coliseum (1995-2019)	Bermuda Grass	Grass	Yes (since 2020)
Los Angeles Chargers	Inglewood, CA (2017-present) San Diego, CA (2010-2016)	Sofi Stadium (2020-present) Dignity Health Sports Park (2017-2019) Qualcomm Stadium (1967-2016)	Hellas Matrix Turf (2020-present) Bermuda Grass (1967-2019)	*^*b*^*	Yes, not fully enclosed (since 2020)
Miami Dolphins	Miami Gardens, FL	Hard Rock Stadium *^*b*^* Formerly Joe Robbie Stadium, Pro Player Stadium, Dolphin Stadium, Land Shark Stadium, Sun Life Stadium	Bermuda Grass	Grass	No
Minnesota Vikings	Minneapolis, MN	US Bank Stadium (2016-present) TCF Bank Stadium (2014-2015) Metrodome (1982-2013)	Global Artificial Turf, Speed S5 (2016-present) FieldTurf (2014-2015) UBU Speed S3 (2011-2013) Sportexe Momentum (2010)	Turf	Yes (2016-present) No (2014-2015) Yes (2011-2013)
New England Patriots	Foxborough, MA	Gillette Stadium	Field Turf	Turf	No
New Orleans Saints	New Orleans, LA	Caesars Superdome *^*b*^* Formerly Louisiana Superdome, Mercedes-Benz Superdome	Turf Nation S5	Turf	Yes
New York Giants, Jets	East Rutherford, NJ	MetLife Stadium	FieldTurf (2023) UBU Speed S5 (2010-2022)	Turf	No
Philadelphia Eagles	Philadelphia, PA	Lincoln Financial Field	Bermuda Grass	Grass	No
Pittsburgh Steelers	Pittsburgh, PA	Acrisure Stadium *^*b*^* Formerly Heinz Field	Kentucky Bluegrass	Grass	No
San Francisco 49ers	Santa Clara, CA	Levi’s Stadium (2014-present) Candlestick Park (1971-2013)	Bermuda Grass	Grass	No
Seattle Seahawks	Seattle, WA	Lumen Field *^*b*^* Formerly Seahawks Stadium, Qwest Field, Century Link Field	FieldTurf	Turf	Roofed, not fully enclosed
Tampa Bay Buccaneers	Tampa, FL	Raymond James Stadium	Bermuda Grass	Grass	No
Tennessee Titans	Nashville, TN	Nissan Stadium *^*b*^* Formerly Adelphia Coliseum, LP Field	Hellas Matrix Turf (2023-present) Grass (1999-2023)	*^*b*^*	No
Washington Commanders	Landover, MD	Northwest Stadium *^*b*^* Formerly FedEx Field, Jack Kent Cooke Stadium	Bermuda Grass	Grass	No

a NFL, National Football League.

b Indicates playing surface changed between turf and grass over the years.
